# Cortical Topography of Error-Related High-Frequency Potentials During Erroneous Control in a Continuous Control Brain–Computer Interface

**DOI:** 10.3389/fnins.2019.00502

**Published:** 2019-05-22

**Authors:** Nile R. Wilson, Devapratim Sarma, Jeremiah D. Wander, Kurt E. Weaver, Jeffrey G. Ojemann, Rajesh P. N. Rao

**Affiliations:** ^1^Department of Bioengineering, University of Washington, Seattle, WA, United States; ^2^Department of Radiology, University of Washington, Seattle, WA, United States; ^3^Department of Neurological Surgery, University of Washington, Seattle, WA, United States; ^4^Paul G. Allen School of Computer Science & Engineering, University of Washington, Seattle, WA, United States

**Keywords:** brain–computer interface, electrocorticography, error-related potential, error potential, execution error, low-level error

## Abstract

Brain–computer interfaces (BCIs) benefit greatly from performance feedback, but current systems lack automatic, task-independent feedback. Cortical responses elicited from user error have the potential to serve as state-based feedback to BCI decoders. To gain a better understanding of local error potentials, we investigate responsive cortical power underlying error-related potentials (ErrPs) from the human cortex during a one-dimensional center-out BCI task, tracking the topography of high-gamma (70–100 Hz) band power (HBP) specific to BCI error. We measured electrocorticography (ECoG) in three human subjects during dynamic, continuous control over BCI cursor velocity. Subjects used motor imagery and rest to move the cursor toward and subsequently dwell within a target region. We then identified and labeled epochs where the BCI decoder incorrectly moved the cursor in the direction opposite of the subject’s expectations (i.e., BCI error). We found increased HBP in various cortical areas 100–500 ms following BCI error with respect to epochs of correct, intended control. Significant responses were noted in primary somatosensory, motor, premotor, and parietal areas and generally regardless of whether the subject was using motor imagery or rest to move the cursor toward the target. Parts of somatosensory, temporal, and parietal areas exclusively had increased HBP when subjects were using motor imagery. In contrast, only part of the parietal cortex near the angular gyrus exclusively had an increase in HBP during rest. This investigation is, to our knowledge, the first to explore cortical fields changes in the context of continuous control in ECoG BCI. We present topographical changes in HBP characteristic specific to the generation of error. By focusing on continuous control, instead of on discrete control for simple selection, we investigate a more naturalistic setting and provide high ecological validity for characterizing error potentials. Such potentials could be considered as design elements for co-adaptive BCIs in the future as task-independent feedback to the decoder, allowing for more robust and individualized BCIs.

## Introduction

Everyone makes mistakes and can learn from them. But the neurophysiological mechanisms behind how we recognize and use these mistakes to learn is still not completely understood. Prior studies have focused on the error-related potential (ErrP), an event-locked electrophysiological response generated during task rule violations. The vast majority of our understanding to date of the ErrP originates from electroencephalography (EEG) studies (Ferrez and Millan, 2008; Iturrate et al., 2013; Spüler and Niethammer, 2015; Zhang et al., 2015; Kreilinger et al., 2016). The typical coverage and high temporal resolution of EEG, relative to fMRI, allow for the identification of wide-spread voltage changes in response to error. However, because EEG is non-invasive, electrical signals from the cortex attenuate and diffuse as they travel up through the skull, leading to lower signal-to-noise ratio (SNR) and challenges in source localization (Jatoi et al., 2014; Olson et al., 2016). To circumvent some of these limitations, we investigate error-related potentials in a one-dimensional brain–computer interface (BCI) task using subdural electrocorticography (ECoG) in human subjects.

Brain–computer interfaces represent a particularly useful opportunity to characterize error-related brain responses. BCIs rely on closed-loop (typically) visual feedback to inform the user of their control and on-going performance. This feedback is hypothesized to be key to the BCI learning process and performance improvement (Green and Kalaska, 2011), analogous to the utility of somatosensory feedback during the acquisition of new motor skills (Newell, 1991). BCI decoders have traditionally been static, in the sense that initial parameters in the algorithm would be set and only changed by manual updates performed by the BCI technician. Recently, however, there has been a push to develop dynamic feedback systems that automatically update over time based on pre-task parameters (DiGiovanna et al., 2009; Orsborn et al., 2014; Pohlmeyer et al., 2014; Merel et al., 2015). However, most efforts so far rely on knowledge of the task and of actuator kinematics, thus limiting BCI co-adaptation to the research setting and do not allow for automatic updating based on signals generated by the user. A co-adaptive BCI may improve the user experience by promoting faster mastery of the BCI and by allowing longer term use through accounting for changes in the brain due to plasticity.

Our motivation in this report was to gain a better understanding of the electrophysiological signatures of error potentials in BCI and whether this will serve to better inform unsupervised co-adaptive BCIs. Specifically, relying on ErrPs as a feedback source to inform adaptive BCI decoders, rather than on specific task data, will allow for BCI use in less constrained environments.

Previous work suggests there are different types of error-related potentials which manifest in different contexts (Milekovic et al., 2012; Spüler and Niethammer, 2015). Such potentials are generally categorized into two classes, high-level error and low-level error (Krigolson and Holroyd, 2007). Krigolson and Holroyd distinguish the two on temporal disparities. Specifically, low-level errors are those immediately correctable in control, and high-level errors as not immediately correctable, which prevent the achievement of a desired goal (Krigolson and Holroyd, 2007). For example, a reactionary turn of the steering wheel to adjust for an unseen bump in the road would be considered low-level error, and failing to reach your destination would be considered high-level error. High-level error, also called outcome error, is thought to be represented by the error-related negativity (ERN), which is often localized to the medial-frontal cortex (Krigolson and Holroyd, 2007; Wessel, 2012) and is believed to be essential to reinforcement learning (Nieuwenhuis et al., 2004). The reinforcement learning theory of the ERN suggests the error signals are generated in the basal ganglia and propagate to the cortex through the anterior cingulate cortex (ACC). Localization of the cortical error-related potentials to the ACC has been suggested in EEG (Krigolson and Holroyd, 2007; O’Connell et al., 2007) and confirmed through ECoG (Bechtereva et al., 2005).

Low-level error, known as target error (Krigolson and Holroyd, 2007; Krigolson et al., 2008) or execution error (Milekovic et al., 2012, 2013), is believed to be represented by positive deflections originating from the posterior parietal cortex (PPC) following commitment of a behaviorally-defined error (Krigolson and Holroyd, 2007; Ladouceur et al., 2007; Krigolson et al., 2008). Although the exact role of this positive activity over PPC is not completely agreed upon, the extent literature converges on a general hypothesis that the PPC is involved with action conflict monitoring, including movement correction (Falkenstein et al., 2000; Nieuwenhuis et al., 2001; Van Veen and Carter, 2002; Krigolson and Holroyd, 2007).

Various EEG studies have identified and investigated ErrPs in the form of ERN (Nieuwenhuis et al., 2001; Ullsperger and von Cramon, 2006; Ladouceur et al., 2007; Krigolson et al., 2008; Iannaccone et al., 2015), P_E_ (Nieuwenhuis et al., 2001; Ladouceur et al., 2007; Navarro-Cebrian et al., 2016), P300 (Krigolson et al., 2008; MacLean et al., 2015), and other signals (Krigolson and Holroyd, 2007; Ferrez and Millan, 2008; Chavarriaga and Millan, 2010; Kim and Kirchner, 2013; Spüler and Niethammer, 2015).

Here we aimed to expand upon our understanding of ErrPs by bridging EEG efforts and characterizing time-frequency responses through ECoG, cross-referencing evoked power effects to the common cortical-localized sites of evoked response ErrPs. We focus on low-level error and its presentation in the parietal cortex, as clinical requirements of electrode placement often constrain consistent frontal coverage. In addition, low-level error is ultimately more relevant in influencing real-time BCI control on a finer time scale than high-level error, which can only be used to provide feedback on longer time-scales (e.g., once per trial).

A previous ECoG study by Milekovic and colleagues demonstrated the presence of ErrPs across multiple cortical regions in a continuous, overt-movement task in human ECoG (Milekovic et al., 2012, 2013). The researchers observed low-level and high-level ErrPs, described as execution and outcome errors, respectively, in the motor, somatosensory, parietal, temporal, and pre-frontal areas. Here, we utilize ECoG to investigate whether errors induced during a motor-imagery BCI task would also result in the typical ErrP profile. We focused exclusively on high-gamma (70–100 Hz) activity. High frequency broadband power (HBP) is thought to best reflect local activity (Ray et al., 2008; Manning et al., 2009; Miller et al., 2009) and is reliably recorded through ECoG. We are particularly interested in examining local response activity for error-processing across the surface of the human brain. Rather than examining errors resultant from (1) failed trial outcomes, (2) induced error, or (3) unexpected stimuli beyond the user’s control, we took a novel approach by examining naturally occurring errors in the BCI decoder’s performance in a continuous control one-dimensional center-out task.

We hypothesize significant HBP changes in error-related detection cortex. This is built on literature and computational models describing ErrPs as a mismatch between sensory expectation from an efference copy and from actual sensory input (in this case, visual) (Holroyd and Coles, 2002; Nieuwenhuis et al., 2004). This mismatch can be thought of as the sensory discrepancy described in Miall and Wolpert’s forward model, which is the difference between actual sensory feedback and expected sensory feedback from an efference copy (Miall and Wolpert, 1996). By gaining a better understanding of the contribution of HBP to ErrPs, we eventually hope to enable unsupervised reinforcement learning in the BCI decoder allowing for robust co-adaptation and improvement of BCI usability.

## Materials and Methods

### Participants

Three patients with medically intractable epilepsy (mean age: 19.67 years, one male), undergoing clinical seizure monitoring at either Harborview Medical Center or Seattle Children’s Hospital, consented and volunteered to participate in research in accordance with the University of Washington Institutional Review Board (see Table 1 for demographics).

**Table 1:** Subject information and task performance separated by trial type.

ID	Sex	Age	Hemisphere	Control channel	# of trials	Successful up trials per run	Successful down trials per run	Overall trial success	# of up error epochs	# of up correct epochs	# of down error epochs	# of down correct epochs
S1	F	11	Left	Hand	49	20.8%	40.0%	30.6%	6	40	39	13
S2	M	13	Left	Tongue	141	28.6%	39.4%	34.0%	31	59	43	32
S3	F	35	Left	Hand	62	20.0%	34.4%	27.4%	21	27	17	22

### Data Recording and Electrode Localization

The electrocorticogram was acquired from subdural macro-scale grid electrodes (Ad-Tech 8 × 8 platinum, 10 mm contact spacing). Cortical potentials were recorded at 1200 Hz using g.USBamps (GugerTec, Graz, Austria) through the BCI2000 software suite (Schalk et al., 2004). Pre-operative T1 MRI scans were co-registered with post-operative CT scans (SPM8) to allow for individualized electrode localization through BioImageSuite software imaging package (Papademetris et al., 2006) in accordance with previously published reports (Casimo et al., 2016). Each subjects’ electrodes were then normalized to the 1 mm MNI 251 brain coordinate system (Evans et al., 1993) using Freesurfer’s ReconAll for multi-subject analysis (Fischl, 2012) and a secondary transform through FSL FLIRT (part of the FMRIB Software Library – FSL^1^) algorithms. Center value MNI coordinates for each electrode were transformed to Talairach space using the MNI anatomical labeling atlas, and Brodmann area (BA) labels were estimated using the Talairach Daemon Client (Talairach and Tournoux, 1988).

### BCI Task

**FIGURE 1:**
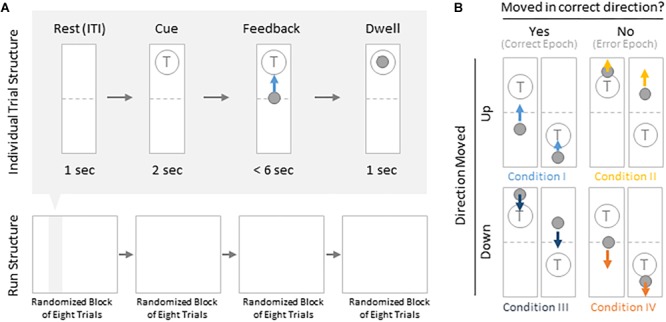
Task structure and epoch conditions. **(A)** Subjects modulated cursor velocity in a one-dimensional center-out BCI task using imagined hand or tongue movement (Table 1) in order to reach and dwell within a target. Cursor was re-centered prior to each trial and was not displayed during the inter-trial interval (ITI) and cue period. Trials were randomized to have the target located above or below the start position, near or far, and small or large. Trials were organized into four blocks, each containing eight randomized trials. **(B)** Within trials, data were binned into epochs based on four movement conditions: cursor moved up correctly (I, light blue), moved up erroneously (II, yellow), moved down correctly (III, navy blue), or moved down erroneously (IV, orange).

Subjects were instructed to control the vertical velocity of a cursor in a one-dimensional center-out BCI task, to reach and dwell within a trial target for 1 s using motor imagery (Figure 1A). Although trial success was determined by whether the cursor dwelled within the target for 1 s, our investigation focuses on correct and erroneous movements made toward or away from the target (specific details provided below). The control electrode was selected through a prior motor screening task, which was used to identify the channel exhibiting the strongest HBP response to a cued imagined movement task of the contralateral hand or tongue (depending on electrode coverage) as previously described (Wander, 2015; Table 1). Consequently the control electrode was always localized to the primary motor cortex.

Each BCI run consisted of four blocks of eight randomly ordered trials. Targets were placed either above or below the starting center point, either large or small (35 or 20% of screen height, respectively) and placed either near or far from the starting center point (20 or 16% of screen height, respectively). This resulted in eight unique trial configurations per block. Each trial was structured to include a 1 s rest period where neither the cursor nor target were displayed [inter-trial interval (ITI)], followed by a 2 s cue period where the target was visible, followed by a feedback period of up to 6 s where subjects would attempt to reach and dwell within the trial target for 1 s. Each trial would terminate either when dwell time was reached or the trial timed out, whichever came first.

For the purposes of these analyses, we grouped trial configurations to only distinguish between trials where the target was placed above or below the starting point, reflecting differences in behavioral task demands.

To drive the cursor up, subjects were required to increase HBP in their control electrode using motor imagery. HBP was estimated using BCI2000’s auto-regressive filter on the preceding 500 ms of data. HBP was normalized to 6 s of pre-trial data using the BCI2000 built-in normalizer, and were linearly mapped to cursor velocity as described in Wolpaw and McFarland (2004). To drive the cursor down, they were instructed to rest. The cursor velocity would update every 40 ms.

### Offline Analysis for Error-Related Potentials

All signal processing and statistical analyses were conducted in MATLAB (MathWorks, Natick, MA, United States) computing environment. For each subject, we performed common average referencing to account for common noise across all channels in the grid. We then removed 60 Hz noise and isolated the high-gamma frequency band activity (HG, 70–100 Hz) using 4^th^ order Butterworth filters (non-causal), and estimated the amplitude envelope of the signals using a Hilbert transform. Power was calculated by taking the absolute square of the analytical amplitude across the full time series. Then the power for each trial was normalized with respect to the preceding ITI (baseline) by calculating the z-score specifically for HBP. The full normalized power time series was smoothed using a sliding Gaussian window with a window width of 40 samples to match the update rate of the task ran in BCI2000.

#### Error and Correct Window Extraction

We were specifically interested in the topography of the responsive HBP during periods of BCI error. To accomplish this, we first grouped subject’s electrodes by identified Brodmann Areas. Second, we defined decoder error as a mismatch between the decoder assessment of HBP and the subject’s goal-directed intention. This was defined operationally as when the slope of the cursor movement (at any junction across the 6000 ms duration of a trial) was in the direction opposite of the target position for a continuous period of 400 ms. This definition allowed for the identification of improper decoding under the assumption that subjects intend to move the cursor toward a target during trial feedback (for clarification, see Figure 1B). We reasoned 400 ms duration is sufficient time for the subjects to realize error during real-time continuous feedback (Gerson et al., 2005). We then identified the beginning of this period as *t* = 0 in error identification. Likewise, correct performance windows were extracted where the cursor movement was in the direction toward the target for a 400 ms period, with *t* = 0 at the start of this period. We then extracted error and correct epochs from these error and correct windows, respectively.

**FIGURE 2:**
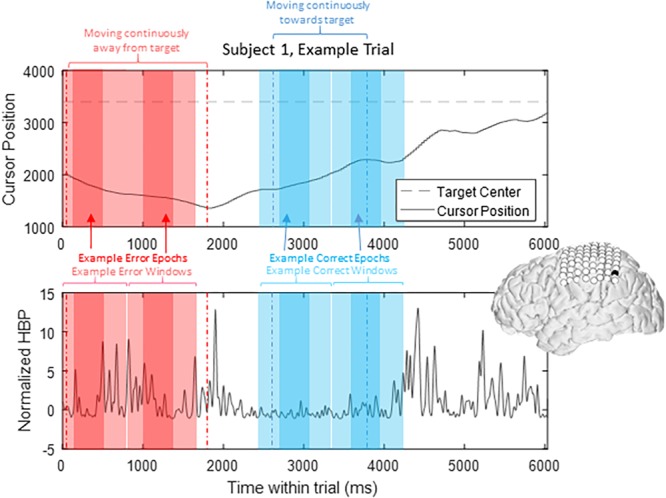
HBP throughout one trial. In this parietal channel in one subject (shown in black), we see a decrease in HBP as the cursor moves correctly toward the target and increase when the cursor is no longer moving as intended. The highlighted light red sections shows example error windows, where their *t* = 0 time points are defined by 400 ms of continuous movement away from the target. The darker red areas represent example error epochs. Likewise, the highlighted light blue sections show example correct windows, and the dark blue sections show example correct epochs, as detailed in Section “Offline Analysis for Error-Related Potentials.”

To prevent overlap between epochs, we extracted only one epoch per window, where we defined windows of 1000 ms starting from 200 ms prior to our *t* = 0 time points to 800 ms after, based on previously published reports investigating error-related potentials in an overt-movement ECoG task (Milekovic et al., 2012). Figure 2 shows data of one full length trial from an example electrode with example windows and example epochs. Note that there are often multiple error and/or correct epochs within any given trial, based on our pre-defined states described below.

From these defined windows, we classify epochs into the four conditions presented in Figure 1B. Specifically, error epochs were classified as when the cursor moves incorrectly upwards when located above a target (Condition II) and when the cursor moves incorrectly downwards when located below a target (Condition IV). Finally, correct epochs were defined as when the cursor moves correctly upwards when located below a target (Condition I) and when the cursor moves correctly downwards when located above a target (Condition III).

#### Statistical Analysis Epochs From Windows

To contrast HBP behavior across the cortical sampling space during real-time continuous error detection, we generated statistical analysis epochs from error and correct windows. These epochs were defined as the samples from *t* = 100–500 ms in their respective 1000 ms windows (where *t* = 0 corresponds to the start of 400 ms consecutive movement in one direction, as described above). Previous ErrP work by Milekovic et al. (2012) observed that the window from 100 to 800 ms after error onset engendered ECoG, error-related components during an overt motor control task. We used the length and range of *t* = 100–500 ms after error onset to characterize responsive HBP behavior based on (1) a relatively short, continuous trial period (6000 ms) and (2) previous EEG observations of typical higher-order processing time of visual cues ranging from 150 to 500 ms, depending on the cortical area being examined (Gerson et al., 2005).

### Statistical Analysis

At the group analysis level, we conducted a two-way ANOVA by extracting mean HBP from our defined statistical analysis epochs and estimating main effects of trial type (whether the target was located above or below the center starting position, requiring motor imagery or rest, respectively) and performance [whether the epoch was a correct epoch (Conditions I and III) or an error epoch (Conditions II and IV)] on HBP for each Brodmann Area available.

We utilized *post hoc* two-sample *t*-tests (FDR corrected) to identify significant interactions of correct and erroneous decoder behavior epochs by subject action type (active motor imagery or rest). We present our findings through exploring Error-related Potentials as changes in HBP across cortical areas. That is, our *post hoc* approach compares (1) HBP of all error epochs and (2) HBP of all correct epochs from all channels falling within each Brodmann Area of interest. Finally, at the individual level, we utilized these two-sample *t*-tests.

## Results

### Task Performance

As common with motor imagery controlled BCIs, the users experienced difficulty in achieving high task performance without an extensive calibration period (Wolpaw and McFarland, 2004; Blankertz et al., 2007, 2008, 2010). The low overall trial success of the subjects (average trial success 30.67%, Table 1) may be due to the difficulty of the task requirement to dwell within the target, and the limited amount of time we had with each subject for training (Table 1). Overall, all three subjects had greater trial success when the target was below the cursor starting position (average trial success 37.93%, Table 1). The effects of task performance on error potentials is discussed in Section “Discussion.”

### Effect of Trial Type and Performance on Group HBP Responses

To determine the HBP response topography of error performance (whether the cursor moved accurately toward or away from the target) we conducted a two-way ANOVA on HBP across BA regions. Results from all available BA regions are presented in Supplementary Table 2. Here we focus on specific BA regions of interest related to ErrPs. Supplementary Table 1 denotes the number of contributing electrodes from each subject within each BA investigated.

A significant main effect of trial type was observed in BA 4 [primary motor cortex – *F*(1,999) = 4.49, *p* = 0.0343], BA 6 [premotor cortex – *F*(1,4258) = 14.01, *p* = 0.0002], BA 40 ([inferior parietal lobule – *F*(1,4918) = 7.21, *p* = 0.0073], and BA 43 [*F*(1,161) = 7.59, *p* = 0.0065]. A significant main effect of performance was observed in BA 3 [primary somatosensory cortex, *F*(1,912) = 5.92, *p* = 0.00152], BA 40 [*F*(1,4918) = 4.48, *p* = 0.0342], and BA 4 [*F*(1,999) = 4.49, *p* = 0.0343]. Importantly, we noted a statistically significant interaction between the trial type, control requirement and performance in primary somatosensory cortex [BA 3; *F*(1,912) = 3.97, *p* = 0.0466], in primary motor cortex [BA 4; *F*(1,999) = 8.46, *p* = 0.0037] as well in the inferior parietal cortex [BA 40; *F*(1,4918) = 6.09, *p* = 0.0136]. For all ANOVA results, please refer to Supplementary Table 2.

### Error-Related HBP Time Series by Brodmann Areas

**FIGURE 3:**
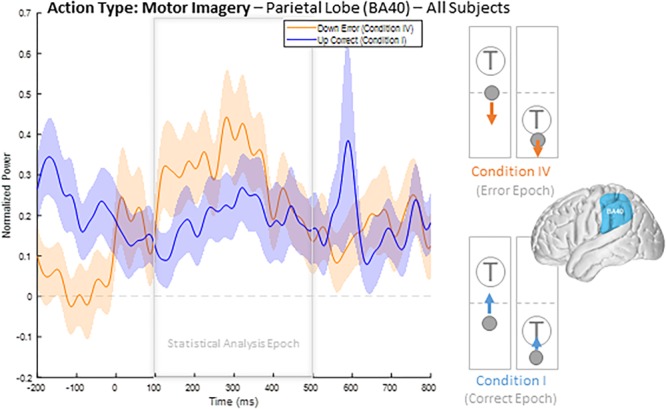
Time series of mean HBP during motor imagery in the decoder error and correct conditions in the parietal lobe (BA 40, highlighted in the brain inset). In these conditions, the subjects were attempting to move the cursor toward the target through eliciting motor imagery. In the erroneous condition, the BCI mistakenly decoded the subject’s intention as wanting to move downwards with rest. Error onset begins at time *t* = 0 ms for the Condition IV plot. Statistical analyses were performed using the time window of *t* = 100–500 ms (i.e., the statistical analysis window), as indicated by the window on the figure. Shaded region shows standard error of the mean. Dashed gray line represents baseline.

To illustrate our overall HBP response profiles, we plotted the mean time-series for all four epoch conditions generated by averaging the responses of all constituent electrodes from all subjects for significant BA regions. Figure 3 shows the mean time-series during the correct and error windows used to extract our Conditions I and IV epochs in all electrodes placed over the inferior parietal lobule (BA 40). We observed increased HBP after error onset at *t* = 0 ms (red) when the decoder failed to recognize the subject’s motor imagery as intent to move the cursor upwards toward the target (Condition IV). Contrarily, we did not see a general increase in HBP when the decoder was correctly decoding the subject’s motor imagery (Condition I). During rest, we did not see a change in HBP relative to error onset (Supplementary Figure 1). We generated similar plots for all available BAs during both motor imagery and during rest. Note, that *t* = 0 ms is a window-unique classification based on our behavioral mismatch between cursor trajectory and decoder output. Our *t* = 0 is not a phase-resetting, evoked boundary event in the classic sense of evoked potentials. Importantly, because there were typically multiple error and correct epochs within any given trial, *t* < 0 reflects behaviorally heterogeneous conditions.

Collectively, this approach provides a useful description of the overall responsive cortical regions generating ErrPs. We next used *post hoc* tests to determine the specific nature of HBP activity as a function of error and correct condition type. We contrasted two different populations for a given action type, motor imagery or rest for significantly responsive regions: (1) the mean value for each error epoch 100–500 ms following error onset from all electrodes within the specified BA, and (2) the mean value for each epoch during correct decoder performance 100–500 ms following the start of recognized correct performance, from all electrodes within the specified BA. We applied a one-sided *t*-test to test the specific hypothesis that HBP is greater in error than in correct epochs.

**FIGURE 4:**
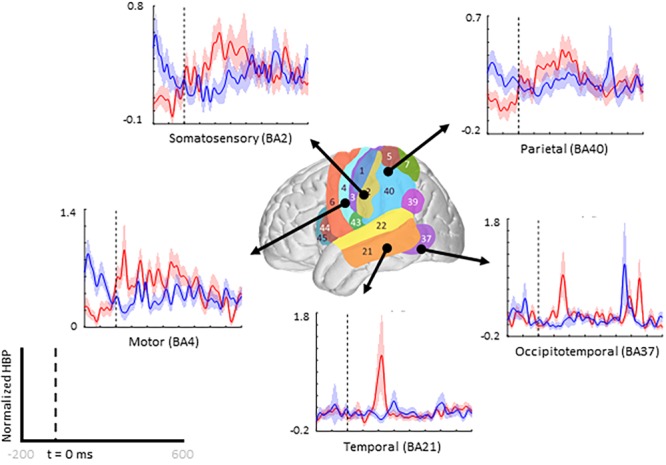
Increased HBP in multiple cortical areas during motor imagery error. The brain in the center shows the spatial range for each Brodmann Area available in our subject population, with each area labeled by their corresponding number. Each plot shows the average response within the specified Brodmann Area during erroneous decoding (red) and during correct decoding (blue), 100 to 500 ms after error onset (indicated by the vertical dashed line).

When comparing average responses following error onset (100–500 ms) during motor imagery (Condition IV–Condition I), we found motor, somatosensory, temporal, and parietal areas as having greater HBP in error epochs than in correct epochs (Figure 4). Specifically, HBP in Condition IV (motor imagery error) were significantly greater than in Condition I (motor imagery correct) in BAs 4 and 40 (one-sided Student’s *t*-test, FDR-adjusted *p* < 0.05). During rest error (Condition II–Condition III), BA 4 was statistically significant (Supplementary Figure 2).

### Low Frequency Error-Related Potentials by Brodmann Areas

In addition to investigating increases in HBP in error epochs as compared to correct epochs, we also observed increases in spectral power in lower frequency bands in these same conditions. Although lower frequency activity is not as localized as high-gamma activity, some lower frequency bands have played an important role in ErrP investigations in EEG work (Trujillo and Allen, 2007; Atchley et al., 2017; Glazer et al., 2018).

Like with HBP, we compared the band power between 100 and 500 ms after error onset and correct performance using one-sided Student’s *t*-tests (alpha = 0.05) and correcting for multiple comparisons using FDR-adjusted *p*-values. For the delta band (<4 Hz), we observed significantly greater power in error epochs compared to in correct epochs, regardless of movement direction, posterior to the temporoparietal junction (BA 39). For the theta band (4–8 Hz), we observed significantly greater power in error epochs compared to in correct epochs, regardless of movement direction, in Brodmann Area 9 (frontal) and in the temporal lobe (BAs 21, 22, and 37). For the alpha band (8–13 Hz), we observed significantly greater power in error epochs compared to in correct epochs, regardless of movement direction, posterior to the temporoparietal junction (BA 39) and in the temporal lobe (BAs 21 and 22). Lastly, for the beta band (13–30 Hz), we observed significantly greater power in error epochs compared to in correct epochs, regardless of movement direction, only in the temporal lobe (BA 21). For a full table of *t*-test results for all available Brodmann Areas and bands, see Supplementary Table 3.

### Error-Related Potentials in Individual Subjects

**FIGURE 5:**
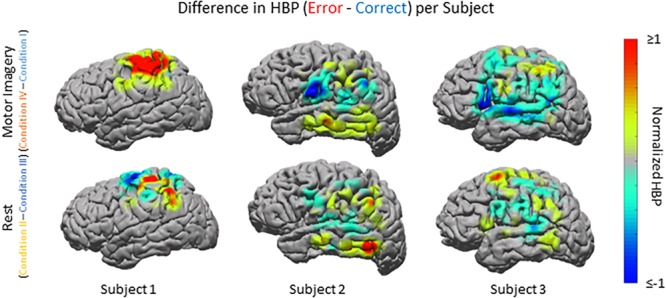
HBP during correct and erroneous BCI decoder performance per individual subject. Difference in high-gamma power during correct and error epochs 100–500 ms after error onset for each subject. Top row shows power of Condition IV–Condition I, bottom row shows power of Condition II–Condition III. Heat maps were scaled to visualize the most robust effects.

Beyond region of interest event-related error analysis, we also explored individual electrode response topography for each subject. Contributions from each electrode are presented in Figure 5 as the difference in mean HBP 100–500 ms following error onset in erroneous and correct decoding, during motor imagery. To visualize this topography, we used a Gaussian spatial smoothing kernel across electrodes allowing for the visualization of cortical-response ‘heat maps’ at the individual level. Warm colors indicate a positive difference where HBP during error is greater than HBP during correct decoding.

**FIGURE 6:**
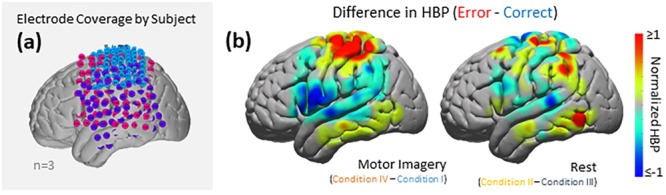
HBP during correct and erroneous BCI decoder performance. **(a)** Electrode coverage by subjects in the left (*n* = 3), separated by color (Subject 1 in blue, Subject 2 in purple, Subject 3 in pink). **(b)** Difference in high-gamma power during correct and error epochs 100–500 ms after error onset. Left shows power of Condition IV–Condition I, right shows power of Condition II–Condition III. Heat maps were scaled to visualize the most robust effects.

As seen in Figure 5, 6A, electrode coverage per subject varies thus yielding variable number of electrodes per Brodmann Area (Supplementary Table 1). Similar to the group-wide analysis, we also determined significance of BAs within individual subjects by comparing the respective error and correct epochs applying one-sided Student’s *t*-tests (alpha = 0.05) and correcting for multiple comparisons using FDR-adjusted *p*-values.

For Subject 1 (11 years old), the majority of electrodes present within the following areas had statistically significantly greater HBP during error than during correct in the motor imagery case (Condition IV–Condition I): BAs 1, 3, 5–7, and 40 (one-sided Student’s *t*-test, FDR-adjusted *p* < 0.05). Brodmann Areas 2 and 4 had a few significant electrodes. There was at least one significant electrode for all observable areas in this subject during motor imagery. During rest (Condition II–Condition III), the number of significant electrodes per respective area was lower than during motor imagery, except for in BA 4. Like during motor imagery, there was always at least one electrode per area that was significant.

For Subject 2 (13 years old), 50% or more of electrodes present within the following areas had statistically significantly greater HBP during error than during correct in the motor imagery case: BAs 2–4, 9, 21, 37, 40, and 42 (one-sided Student’s *t*-test, FDR-adjusted *p* < 0.05). Brodmann Area 6 had one significant electrode, BA 22 had three significant electrodes, and BAs 39 and 43 did not have any. During rest, the number of significant electrodes per respective area was typically lower than during motor imagery. Some areas, which had most of their electrodes significant during motor imagery, do not have any significant differences during rest (BAs 3–4, 9, 42).

For Subject 3 (35 years old), 50% or more of electrodes present within the following areas had statistically significantly greater HBP during error than during correct in the motor imagery case: BAs 4 and 5 (one-sided Student’s *t*-test, FDR-adjusted *p* < 0.05). Brodmann Areas 1, 6, 21, and 40 had at least one significant electrode each, and areas 2, 3, 7, 9, 22, 39, 42, 44, and 45 had no significant differences. During rest, the number of significant electrodes per respective area was typically higher than during motor imagery. With the exception of BA 7, all the areas which had no significant electrodes during motor imagery had at least one significant electrode during rest.

### Group Analysis: Cortical Topography of Error-Related HBP Responses

Using the data from each electrode of all subjects (Figure 5), we generated cortical heat maps to observe the overall activity of the group. Figure 6 serves to show the contributions by electrodes instead of presenting the mean response of any given Brodmann Area. As seen when we project each subject’s electrodes onto the MNI brain, each subject has different coverage and therefore contributes a different number of electrodes to each area of interest.

The areas with the most common coverage were BA 40 (part of the parietal cortex) and BA 6 (posterior-most part of the frontal cortex), with 42 and 38 total electrodes per area, respectively. Areas 2–4 also had common coverage but had 10 electrodes or less per area.

**FIGURE 7:**
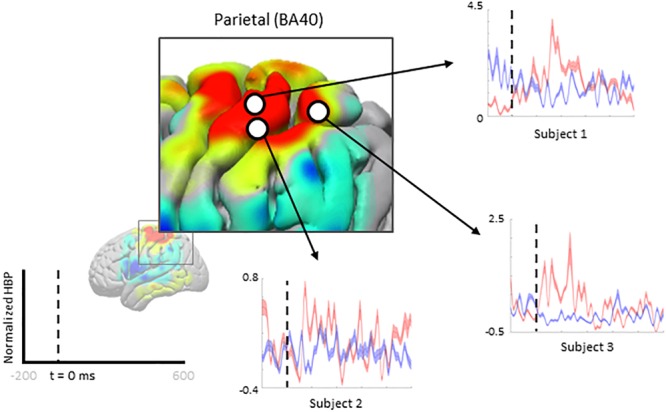
Increased HBP in multiple cortical areas during motor imagery error (parietal). Each plot shows the average response of a single electrode (from one subject, each) within Brodmann Area 40 during erroneous decoding (red) and during correct decoding (blue).

We zoom-in to a portion of the parietal lobe in Figure 7 to show examples of individual electrode contributions from all subjects in BA 40, one of the few areas with multiple electrodes from each subject.

## Discussion

We present the brain topography of HBP changes associated with error processing in the context of visual feedback, closed-loop, motor-imagery BCI. Our novel approach to extracting epochs of behaviorally defined error within a free-running BCI context is likely to be more reflective of naturalistic error processing, provides high ecological validity and is specifically relevant to contemporary co-adaptive BCI design. That is, our *post hoc* identification of error epochs, based on violations of intention, circumvent limitations of artificially-induced error events which do not take subject intention into account. To this end, our results are in agreement with a previous study investigating error-related potentials in ECoG in an overt movement task (Milekovic et al., 2012).

Our BCI task involved using both active motor imagery and rest to control the vertical velocity of a cursor to reach and dwell within a target. We investigated the involvement of different Brodmann Areas and individual electrodes across subjects for these two different control paradigms when the decoder incorrectly decodes the subject’s intention and moves the cursor away from the target.

### Interpreting Effects of Trial Type and Performance on HBP

The interaction of trial control requirement (whether the subject needed to bring the cursor up to a target placed above the center starting position using motor imagery) and performance (whether the cursor moved correctly toward or erroneously away from the target) had a statistically significant interaction on HBP in Brodmann Areas 3, 4, 40, and 43. In other words, the difference in HBP between correct performance and erroneous performance were affected by whether motor imagery or rest was required as the initial action to reach the target in the trial. The specificity of this significant interaction was clarified by *post hoc t*-test results for the motor imagery conditions and the rest conditions.

Importantly, we noted a statistically significant effect of trial control requirement on HBP in the primary motor cortex (BA 4) and premotor cortex (BA 6), providing internal validity for our statistical approach. Specifically, the control electrode, located in primary motor cortex, moved the cursor up or down through increasing or decreasing HBP.

### Broader Response Observed for Motor Imagery Error Than for Rest Error

Overall, more areas of the cortex exhibited significantly greater HBP during error in motor imagery cursor control rather than in rest cursor control. BA regions which showed more significant HBP changes in both the motor imagery and rest cases, when including all subjects, were the somatosensory (BAs 1 and 5), motor (BA 4), and parietal (BA 7) cortices. Areas which were exclusively significant during motor imagery include part of somatosensory (BAs 2 and 3), temporal (BAs 21 and 37), and parietal (BA 40) cortices. The only area which was significant during rest but not motor imagery was near the angular gyrus in the parietal lobe (BA 39). Note that we did not have much frontal coverage from any of the subjects, preventing investigation of common areas of interest associated with outcome (not execution) error, such as the ACC (Figure 6A). In Milekovic and colleagues overt movement ECoG study, ErrPs in the motor, somatosensory, parietal, temporal, and pre-frontal areas were observed when an execution error was induced in the subject’s joystick control (Milekovic et al., 2012, 2013).

ErrPs in the motor and somatosensory areas are not unexpected considering they are directly involved in the control and immediate feedback associated with the control. Involvement of other areas may not be as obvious. Previous work has suggested that the parietal lobe is involved with low-level error processing, which cursor control error can be considered (Krigolson and Holroyd, 2007). Although not traditionally explored for error analyses, as there is typically a focus on the ACC and other frontal areas, previous fMRI work suggests the temporal lobe is also involved in error processing (Stevens et al., 2009).

### Corrective Movement

The extent literature also suggests the parietal lobe may be involved in the execution of corrective movements in response to error or low-level error (Calhoun et al., 2006; Krigolson et al., 2008; Navarro-Cebrian et al., 2016). It is of note that the temporoparietal junction (TPJ; BA 40) only had significant HBP during motor imagery error, but the more posterior and superior area of the parietal lobe (BA 7) had significant HBP during both motor imagery and rest error conditions. Krigolson et al. (2008) suggest that low-level errors are mediated in the PPC, which may be reflected by the increased HBP in BA 7 for both error conditions. Interestingly, the same group showed that a P300 response from the TPJ would immediately precede corrective movements in response to error in an earlier study (Krigolson and Holroyd, 2007). Instead of being directly responsible for the corrective movement, Krigolson et al. (2008) postulate that the P300 indicates the updating of an internal model of the task at hand. Navarro-Cebrian et al. (2016) instead suggest the P_E_ from the parietal lobe indicates that enough error information has been gathered to make a decision to change motor output in order to correct for the error. In our current work, the TPJ had significant increases in HBP during motor imagery error, but not during rest error. This may imply that the parietal lobe elicits a greater response when the corrective action to take requires an increase or change in motor output, in this case, increased motor imagery. Rest error may not have resulted in increased local activity in the TPJ because the corrective action to take would be to suppress motor imagery.

### Low Performance and Error Elicitation

Error-related potentials are typically investigated in settings where instances of correct performance greatly outnumber the instances of error. However, recent work by Pezzetta et al. (2018) reverses the correct/error ratio by inducing error events for 70% of the task. In their study, Pezzetta et al. (2018) find that error-related potentials are still elicited even when error occurs during the majority of the task, confirming that the performance-monitoring system engages in the presence of error and not just in the presence of uncommon stimuli.

The subjects of this present investigation had relatively low task performance, with an average trial success rate of 30.67% (Table 1). This low trial success rate can be partially explained by task difficulty and/or BCI task novelty, as the targets would be placed at varying distances from the center and would also vary in size. In particular, the success condition of having to dwell within the target made the task more difficult than similar one dimensional cursor control tasks, such as the Right-Justified Box task. Note that our behavioral definition of low-level error is not dependent on trial success, but instead on successful cursor movements within each trial. Overall, subjects performed marginally better on trials where the target was placed below the cursor starting position (Table 1). Even with this generally poor performance, we believe our investigation to still be valid as error-related potentials are still elicited in tasks where the majority of actions are erroneous (Pezzetta et al., 2018).

### Impact of Age on Error Potentials

Two of the subjects in this investigation were early adolescents of the ages 11 and 13 years old (Subjects 1 and 2, respectively), and the other subject was 35 years old (Subject 3) (Table 1). Human brain maturation from childhood to adulthood is characterized by changes in the structure of and activation of various brain structures, including in the ACC in the prefrontal cortex (Casey et al., 1997; Adleman et al., 2002), a structure essential to conflict monitoring.

Previous work by Ladouceur et al. (2007) found that with a more developed ACC, adults (19+ years old) and late adolescents (14–18 years old) had stronger ERN responses than early adolescents (9–13 years old), however, the P_E_ responses did not differ significantly between any of the groups.

Although most subjects exhibited greater HBP in more areas during motor imagery error, Subject 3 had more electrodes with significantly greater HBP differences between error and correct in rest rather than in motor imagery. While this does not seem to be directly related to the aforementioned developmental changes, it is still possible that error presentation in Subject 3 differed from the younger subjects due to processes related to cortical maturation.

### Error Presentation in the Time Domain

Unlike with well-established error potentials in EEG, which are often measured as particular phase-locked negative and positive deflections in the time domain, we explored ErrPs related to specific changes in the band-limited time-frequency domain due to the high temporal/spatial resolution inherent to ECoG. Our use of a *post hoc* defined behavioral marker for detecting error-onset instead of a controlled, elicited error in control may have also led to less distinct, non-event locked ErrP waveforms. As discussed, analyses in the frequency domain do not provide a clean time-stamped waveform present in multiple electrodes in or across any of the subjects. The higher spatial resolution of ECoG, in addition to our unique epoch boundary markers were determined, contributed to the difficulty of relying on time-domain data for ErrP identification in this study.

Regardless, we attempted to compare topographical results more directly with EEG literature by investigating changes in the raw voltage potentials recorded per channel per subject in all conditions. Due to the nature of our task not having an experimentally controlled induced onset of error, we did not expect, nor did we see, as robust a response as in EEG. We only saw significantly greater voltage amplitude during error than during correct in a few select electrodes in two subjects.

### Implications for BCI

Although this investigation focused on identifying cortical error-related potentials *post hoc*, online classification of error-related potentials have been demonstrated in a few EEG studies (Iturrate et al., 2015; Zander et al., 2016; Cruz et al., 2018). With online ErrP monitoring, future cortical BCI can infer BCI performance without explicit task information, allowing for automatic adaptation of the system based on estimated performance. The task-independent nature of this method could allow for robust adaptive systems that allow for long-term use of BCI that account for changes in recorded brain signals over time.

As this was a preliminary investigation into error-related potentials in cortical BCI, we did not employ online classification methods. The methods presented here could be adapted to work for online classification by continually calculating HBP via a sliding window, and setting a threshold for channels located on particular regions of interest, such as over BA 7. The baseline may be set as the data prior to the start of the sliding window, of a length longer than the sliding window itself. Alternatively, a machine learning model could be developed and trained to classify error and non-error signals and fed the necessary sliding window information for continual classification.

## Conclusion

In this study, we examined the cortical activity of human subjects during a one-dimensional center-out BCI task and investigated how different areas of the cortex behaved during erroneous BCI decoding versus during correct performance. Of all the cortical areas available for analysis, the somatosensory (BAs 1 and 5), motor (BA 4), and the parietal lobe (BA 7) showed significantly greater HBP 100–500 ms after error onset than during correct behavior, regardless of whether the subject was actively imagining movement or resting to achieve their goal. During motor imagery, parts of the somatosensory (BAs 2 and 3), the temporal lobe (BAs 21 and 37), and part of the parietal cortex (BA 40) were exclusively significant. During rest, only part of the parietal cortex near the angular gyrus (BA 39) was exclusively significant. Overall, more areas were involved in error processing during the motor imagery error cases rather than during rest error, although there were differences between subjects, with one subject having more significant electrodes during rest. The observed activity of these areas agrees with previous work suggesting the involvement of the parietal and temporal areas in error processing.

Although our results generally agree with previous work performed with overt movement in ECoG (Milekovic et al., 2012), our error-related potentials (ErrPs) do not present themselves as the well-defined waveforms discussed in the EEG literature, likely due to the diffuse timing of our error events. The presence of ErrPs in both overt- and imagined-movement controlled ECoG tasks suggests error processing is impartial to the method of control in a task.

Our investigation is the first to explore ErrPs in the context of continuous control in a cortical BCI. As the BCI field delves further into understanding error and reinforcement learning, it is critical that we understand error processing at various spatial and temporal levels in a multitude of conditions. This study contributes to the field by focusing on continuous control (instead of discrete control for simple selection) representing a more naturalistic setting for characterizing error potentials in the brain. Additionally, we report the first description of the responsive local high-frequency activity using high-gamma band power in a BCI, instead of more global signals such as theta band activity in EEG.

In addition to exploring error processing in the context of motor BCI, we are also interested in the effects of different forms of feedback during continuous control, not just visual (as is typical with most current BCIs). In the future of BCIs and their adoption into neuroprostheses, we will need to understand the effect of other forms of feedback, which inform volitional control, on BCI learning. Ultimately, the use of ErrPs as an automatic feedback signal to future BCIs will allow for co-adaptation, leading to better and longer-lasting control. Greater performance and longer ability of use will allow these co-adaptive BCIs to break out of the confines of the research setting and make their way into clinics and home settings.

We would like to explore the long-term effects of learning on the error-related potentials, but our limited time with research subjects renders this nearly impossible. A better understanding of ErrPs and their usability over time is crucial for implementing co-adaptive BCI systems which rely on ErrPs for feedback. Longer use in the experimental setting may allow for the development of robust classification techniques which can assist in real-time error detection in the future.

## Ethics Statement

This study was carried out in accordance with the protocol approved by the Seattle Children’s Hospital Institutional Review Board for Subjects 1 and 2, and of the University of Washington Institutional Review Board, Human Subjects Division, Committee K for Subject 3. All subjects, or their legal guardians if under age 18, gave written informed consent in accordance with the Declaration of Helsinki.

## Author Contributions

NW performed the analyses and wrote the manuscript. DS and KW provided input and mentorship through the analysis and writing. JW designed the task and collected the data. JO and RR supervised the work.

## Conflict of Interest Statement

The authors declare that the research was conducted in the absence of any commercial or financial relationships that could be construed as a potential conflict of interest.

## Footnotes

1 www.fmrib.ox.ac.uk/fsl
