# Supplementary material for: Cortical Topography of Error-Related High-Frequency Potentials During Erroneous Control in a Continuous Control Brain–Computer Interface
Source: Front Neurosci. 2019 May 22;13:502. doi: 10.3389/fnins.2019.00502 (PMC6541115; doi:10.3389/fnins.2019.00502)
Supplement: Supplementary file 1 [file Data_Sheet_1.docx]

Supplementary Material

Cortical topography of error-related high-frequency potentials during erroneous control in a continuous control Brain-Computer Interface

Nile R. Wilson*, Devapratim Sarma, Jeremiah D. Wander, Kurt E. Weaver, Jeffrey G. Ojemann, and Rajesh P. N. Rao

*** Correspondence:** Nile Wilson: [nilew@uw.edu](mailto:nilew@uw.edu)


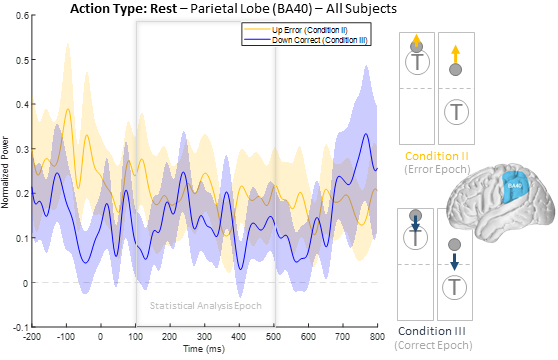


**Supplementary Figure 1.** Time series of mean HBP during rest in the decoder error and correct conditions in the parietal lobe (BA40, highlighted in the brain inset). In these conditions, the subjects were attempting to move the cursor towards the target through rest. In the erroneous condition, the BCI mistakenly decoded the subject’s intention as wanting to move upwards with motor imagery. Error onset begins at time t = 0 ms for the Condition II plot. Statistical analyses were performed using the time window of t = 100-500 ms, as indicated by the window on the figure. Shaded region shows standard error of the mean. Dashed gray line represents baseline.

| **Supplementary Table 1.** Number of electrodes per Brodmann Area. | | | | | | | | | | | | | | | | | | |
| --- | --- | --- | --- | --- | --- | --- | --- | --- | --- | --- | --- | --- | --- | --- | --- | --- | --- | --- |
| Brodmann Area | | 1 | 2 | 3 | 4 | 5 | 6 | 7 | 9 | 21 | 22 | 37 | 39 | 40 | 42 | 43 | 44 | 45 |
| Number of Electrodes | Subject 1 | 4 | 3 | 5 | 5 | 5 | 16 | 6 | 0 | 0 | 0 | 0 | 0 | 16 | 0 | 0 | 0 | 0 |
|  | Subject 2 | 0 | 4 | 1 | 1 | 0 | 10 | 0 | 2 | 11 | 8 | 4 | 1 | 14 | 2 | 1 | 0 | 0 |
|  | Subject 3 | 3 | 1 | 3 | 4 | 1 | 12 | 3 | 1 | 5 | 9 | 0 | 2 | 12 | 2 | 0 | 3 | 2 |
|  | Total | 7 | 8 | 9 | 10 | 6 | 38 | 9 | 3 | 16 | 17 | 4 | 3 | 42 | 4 | 1 | 3 | 2 |


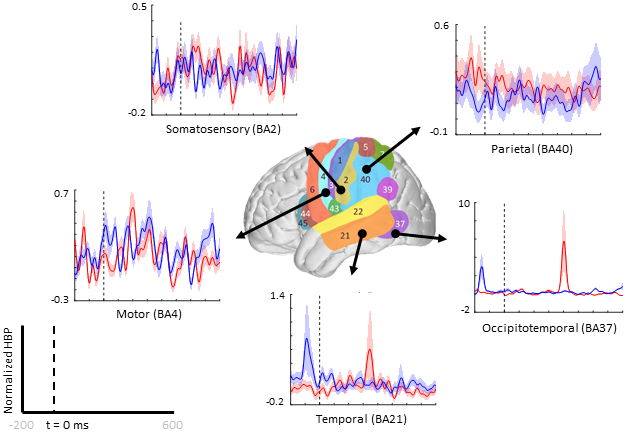


**Supplementary Figure 2.** Increased HBP in multiple cortical areas during rest error. The brain in the center shows the spatial range for each Brodmann Area available in our subject population, with each area labeled by their corresponding number. Each plot shows the average response within the specified Brodmann Area during erroneous decoding (red) and during correct decoding (blue), 100 ms to 500 ms after error onset (indicated by the vertical dashed line).

| **Supplementary Table 2.** Resultant p-values for the two-way ANOVA estimating the main effects of trial type (whether the target was located above or below the center starting position, requiring motor imagery or rest, respectively) and performance (whether the epoch was a correct epoch (Conditions I and III) or an error epoch (Conditions II and IV)) on HBP for each Brodmann Area available. Values below alpha of 0.05 are highlighted in green. | | | | |
| --- | --- | --- | --- | --- |
| Brodmann Area | Subjects Included | ANOVA results (interaction) | ANOVA results (trial type) | ANOVA results (performance) |
| 1 | Subjects 1, 3 | F(1, 649) = 1.65, p = 0.1999 | F(1, 649) = 2.44, p = 0.1187 | F(1, 649) = 2.31, p = 0.1288 |
| 2 | All | F(1, 1037) = 2.69, p = 0.1011 | F(1, 1037) = 2.59, p = 0.1078 | F(1, 1037) = 2.3, p = 0.1296 |
| 3 | All | F(1, 912) = 3.97, p = 0.0466 | F(1, 912) = 3.76, p = 0.0529 | F(1, 912) = 5.92, p = 0.0152 |
| 4 | All | F(1, 999) = 8.46, p = 0.0037 | F(1, 999) = 4.49, p = 0.0343 | F(1, 999) = 2.49, p = 0.115 |
| 5 | Subjects 1, 3 | F(1, 573) = 2.48, p = 0.1157 | F(1, 573) = 3, p = 0.0836 | F(1, 573) = 9.61, p = 0.002 |
| 6 | All | F(1, 4258) = 0.08, p = 0.775 | F(1, 4258) = 14.01, p = 0.0002 | F(1, 4258) = 1.93, p = 0.1648 |
| 7 | Subjects 1, 3 | F(1, 845) = 2.73, p = 0.0987 | F(1, 845) = 0.28, p = 0.5981 | F(1, 845) = 3.42, p = 0.0649 |
| 9 | Subjects 2, 3 | F(1, 413) = 0.24, p = 0.6209 | F(1, 413) = 3.01, p = 0.0833 | F(1, 413) = 3.92, p = 0.0485 |
| 21 | Subjects 2, 3 | F(1, 2246) = 0.12, p = 0.7312 | F(1, 2246) = 0.26,p = 0.6104 | F(1, 2246) = 2.54, p = 0.1112 |
| 22 | Subjects 2, 3 | F(1, 2099) = 0.76, p = 0.3833 | F(1, 2099) = 2.86, p = 0.0909 | F(1, 2099) = 18.69, p = 0 |
| 37 | Subject 2 | F(1, 656) = 1.62, p = 0.203 | F(1, 656) = 0.82, p = 0.3642 | F(1, 656) = 1.82, p = 0.1777 |
| 39 | Subjects 2, 3 | F(1, 335) = 0.76, p = 0.3845 | F(1, 335) = 1.31, p = 0.2509 | F(1, 335) = 0.04, p = 0.8385 |
| 40 | All | F(1, 4918) = 6.09, p = 0.0136 | F(1, 4918) = 4.48, p = 0.0342 | F(1, 4918) = 7.21, p = 0.0073 |
| 42 | Subjects 2, 3 | F(1, 500) = 0.08, p = 0.7749 | F(1, 500) = 0.01, p = 0.9105 | F(1, 500) = 0.66, p = 0.4158 |
| 43 | Subject 2 | F(1, 161) = 9.45, p = 0.0025 | F(1, 161) = 9.41, p = 0.0025 | F(1, 161) = 7.59, p = 0.0065 |
| 44 | Subject 3 | F(1, 257) = 1.53, p = 0.2176 | F(1, 257) = 0.09, p = 0.7683 | F(1, 257) = 0.0967 |
| 45 | Subject 3 | F(1, 170) = 0, p = 0.9737 | F(1, 170) = 0.23, p = 0.6301 | F(1, 170) = 3.78, p = 0.0536 |

| **Supplementary Table 3.** Resultant p-values for testing if band power in error epochs (100-500 ms after error onset) was greater than band power in correct epochs using a one-sided student t-test (p-values FDR-adjusted for multiple comparisons, alpha = 0.05) across all subjects. Values below alpha of 0.05 are highlighted in green. | | | | | | | | | | | | | | | | | | | |
| --- | --- | --- | --- | --- | --- | --- | --- | --- | --- | --- | --- | --- | --- | --- | --- | --- | --- | --- | --- |
| Brodmann Area | |  | 1 | 2 | 3 | 4 | 5 | 6 | 7 | 9 | 21 | 22 | 37 | 39 | 40 | 42 | 43 | 44 | 45 |
| FDR-corrected p-values | HG (70-100 Hz) | MI | <0.001 | <0.001 | <0.001 | <0.001 | <0.001 | 0.5939 | <0.001 | 0.5939 | <0.001 | 0.5939 | <0.001 | 0.5939 | <0.001 | 0.5939 | 0.5939 | 0.5939 | 0.5939 |
|  |  | Rest | <0.001 | 0.5939 | 0.5939 | <0.001 | <0.001 | 0.5939 | <0.001 | 0.5939 | 0.5939 | 0.5939 | 0.5939 | <0.001 | 0.5939 | 0.5939 | 0.5939 | 0.5939 | 0.5939 |
|  | Beta (13-30 Hz) | MI | 0.8312 | 0.8312 | 0.8312 | 0.8312 | 0.8312 | 0.8312 | 0.8312 | 0.8312 | <0.001 | <0.001 | 0.8312 | <0.001 | 0.8312 | 0.8312 | 0.8312 | 0.8312 | 0.4655 |
|  |  | Rest | <0.001 | <0.001 | <0.001 | <0.001 | <0.001 | 0.3559 | <0.001 | 0.3559 | <0.001 | 0.3559 | <0.001 | 0.3559 | <0.001 | <0.001 | 0.3559 | <0.001 | 0.3559 |
|  | Alpha (8-13 Hz) | MI | 0.7283 | 0.7283 | 0.7283 | 0.7283 | 0.7283 | 0.7283 | 0.7283 | 0.7283 | <0.001 | <0.001 | 0.7283 | <0.001 | 0.7283 | 0.7283 | 0.7283 | 0.0027 | 0.0326 |
|  |  | Rest | <0.001 | 0.0778 | <0.001 | <0.001 | 0.2947 | <0.001 | 0.2947 | <0.001 | <0.001 | <0.001 | <0.001 | <0.001 | <0.001 | <0.001 | <0.001 | 0.2947 | 0.2947 |
|  | Theta (4-8 Hz) | MI | 0.6532 | 0.6532 | 0.6532 | 0.6532 | <0.001 | 0.6532 | 0.6532 | <0.001 | <0.001 | <0.001 | <0.001 | 0.6532 | 0.6532 | 0.6532 | 0.6532 | <0.001 | 0.6532 |
|  |  | Rest | 0.4747 | <0.001 | 0.4747 | <0.001 | 0.4747 | <0.001 | 0.4747 | <0.001 | <0.001 | <0.001 | <0.001 | <0.001 | <0.001 | 0.4747 | 0.4747 | 0.4747 | 0.4747 |
|  | Delta (<4 Hz) | MI | <0.001 | 0.7124 | 0.7124 | <0.001 | <0.001 | 0.7124 | <0.001 | 0.7124 | 0.7124 | 0.7124 | 0.7124 | <0.001 | 0.7124 | 0.7124 | 0.7124 | 0.7124 | 0.7124 |
|  |  | Rest | 0.4153 | <0.001 | 0.4153 | 0.4153 | 0.4153 | <0.001 | 0.4153 | <0.001 | <0.001 | <0.001 | <0.001 | <0.001 | <0.001 | <0.001 | <0.001 | 0.4153 | 0.4153 |
